# Editorial: Explainable multimodal AI in cancer patient care: how can we reduce the gap between technology and practice?

**DOI:** 10.3389/fmed.2023.1190429

**Published:** 2023-04-13

**Authors:** Michal Rosen-Zvi, Lisa Mullen, Robertus Jan Lukas, Michal Guindy, Maria Gabrani

**Affiliations:** ^1^IBM Research Israel, Haifa, Israel; ^2^Faculty of Medicine, Hebrew University of Jerusalem, Jerusalem, Israel; ^3^Johns Hopkins Medicine, Johns Hopkins University, Baltimore, MD, United States; ^4^Department of Histopathology, Royal Brompton Hospital, London, United Kingdom; ^5^Imaging Services, Assuta Medical Center, Tel Aviv-Yafo, Israel; ^6^IBM Research Zurich, Rüschlikon, Switzerland

**Keywords:** computational medicine, oncology, multimodal data fusion, explainable AI, artificial intelligence (AI)

Cancer is among the most prevalent and complex medical conditions that humanity faces. The typical diagnosis and treatment of patients with cancer includes a diverse medical team ranging from radiologists, pathologists and geneticists to internal medicine experts, surgeons, medical oncologists and radiation oncologists. This team of experts is needed as cancer is a highly heterogeneous condition (1) and the more multimodal and often complex data are collected regarding the specific condition of a patient, the more likely the clinical team will be able to successfully decide on the correct treatment pathway. Indeed, choosing if and how to treat patients who are diagnosed with cancer is challenging as the clinician would like to avoid overtreatment or under treatment but also to ensure the treatment options are tailored to the individual patient and implemented within national cancer pathway timelines. The choice of a treatment approach requires considering not only the immediate side effects but also the known correlation between anticancer treatment such as chemotherapy and the development of secondary cancers (2). Finally, immunotherapy has significantly changed the therapeutic landscape of certain cancers such as lung cancer, but response to (neo)adjuvant treatment is variable, and accurately predicting response remains a challenge (3). To optimize the therapy offered to a patient with cancer, the treatment plan decision-making process often is aided by discussion and consensus opinions of experts in a multi-disciplinary team (a tumor board).

With the advent of AI technologies and their penetration to the clinical world (4), it is expected that AI will help support tumor boards in these challenging decisions. Indeed, in recent years many AI technologies were developed for supporting the diverse medical teams, thereby improving clinical treatment pathways for cancer patients. Taking breast cancer as an example, it has been shown that when AI is applied to the task of cancer detection on mammograms, it can have similar sensitivity to radiologists, can serve as a companion technology and provide a safety net to radiologists in cancer detection (5), and can be used to filter out normal cases to reduce radiologists' workload (6). Similarly, it was shown to be able to serve as companion technology for pathologists (7) and to predict treatment efficacy (8). Most importantly though, the data-driven and evidence-based AI predictions and generated data, allow clinicians to gain new insights and expand their knowledge and understanding of the underlying biology and disease pathogenesis and treatment effectiveness, by providing them with means to further their studies and explorations. However, despite such studies that illustrate the value of AI in assisting physicians, the adoption of AI based technology in clinical practice has been slow. This topic addresses the question of how the gap between technology and practice can be reduced. Note that this topic does not cover non-technical challenges associated with adoption of AI technologies such as who has the responsibility for AI systems' actions and economics barriers for adoption.

The papers published in this topic highlight four barriers to the adoption of AI technologies in cancer patient care. The different papers offer approaches to overcome these limitations. The barriers are:

1. Multimodal analysis—as discussed above, diagnosis and treatment choices in oncology are made based on multiple modalities, from clinical tests that are available in a structured form through medical images and digital pathology to genomic data and more. However, most of the AI studies focus on one modality and hence are inherently limited in their ability to provide accurate support in a decision process or comprehensive insights.
2. Reproducibility of AI algorithms—often AI algorithms are developed in the context of a single data source, one geographic area, a single provider organization, or patients from a single racial/ethnic group. Also, the data used for training and testing the algorithms are often not representative of the cases in the clinic, as strict eligibility criteria are applied, and noisy examples are ruled out. Appropriately heterogeneous and diverse patient populations, together with test datasets are needed to ensure the reproducibility and generalizability of the developed technologies.
3. The black box nature of many of the AI technologies is often used to explain their slow penetration to the clinic. Adding explainability could foster trust in the technology, and support the development and further exploration process by enabling debugging and unbiasing (9).
4. The mapping of decisions on interventions in the clinic to an AI algorithm requires a well-defined goal or set of goals, where the algorithm is designed to identify an optimized solution that achieves the goal(s). AI studies are often driven by data available in a study and hence when choosing the best treatment option, the goals are reduction of recurrence and improved overall survival, as these outcomes are typically documented in the medical record. However, other important treatment outcomes, such as side effects and quality of life, may be ignored.

The papers address the gap between technology and practice focusing on the above barriers. Two papers analyze multimodal data (Prelaj et al., Massafra et al.). A thorough study of how to address noisiness in the data is offered in Mayer et al.. SHapley Additive exPlanations (SHAP) is an explainable technique with solid theoretical background used by two of the papers to support the AI algorithm's finding (Prelaj et al., Massafra et al.). Finally, Solikhah et al. addresses the important question of how cancer survivors define their quality of life (QoL). The paper suggests a culturally suitable, valid, and reliable translation and evaluation of appropriate instrument to measure the QoL of breast cancer patients.

The papers in this topic cover data coming from a broad geographic range, from Italy and Germany to Indonesia. Two papers focus on one of the most prevalent cancers, breast cancer, and two focus on two of the deadliest cancers, lung cancer and ovarian cancer. The papers cover key elements of the patient journey, including diagnosis to treatment efficacy and serve to illustrate and test general approaches of AI technologies applied to multimodal cancer patient data. These papers show that integrating different modalities in an interpretable way enhances cancer understanding and paves the way for personalized patient care.

## Author contributions

All authors listed have made a substantial, direct, and intellectual contribution to the work and approved it for publication.
